# Microglia Responses in Acute and Chronic Neurological Diseases: What Microglia-Specific Transcriptomic Studies Taught (and did Not Teach) Us

**DOI:** 10.3389/fnagi.2017.00227

**Published:** 2017-07-21

**Authors:** Hélène E. Hirbec, Harun N. Noristani, Florence E. Perrin

**Affiliations:** ^1^Institute for Functional Genomics, CNRS UMR5203, INSERM U1191, University of Montpellier Montpellier, France; ^2^Laboratory of Excellence in Ion Channel Science and Therapeutics (LabEx ICST) Montpellier, France; ^3^University of Montpellier, INSERM U1198 Montpellier, France; ^4^École Pratique des Hautes Études (EPHE) Paris, France

**Keywords:** microglia, cell-specific transcriptomics, CNS traumatisms, neurodegenerative diseases, peripheral immune challenges, glioma

## Abstract

Over the last decade, microglia have been acknowledged to be key players in central nervous system (CNS) under both physiological and pathological conditions. They constantly survey the CNS environment and as immune cells, in pathological contexts, they provide the first host defense and orchestrate the immune response. It is well recognized that under pathological conditions microglia have both sequential and simultaneous, beneficial and detrimental effects. Cell-specific transcriptomics recently became popular in Neuroscience field allowing concurrent monitoring of the expression of numerous genes in a given cell population. Moreover, by comparing two or more conditions, these approaches permit to unbiasedly identify deregulated genes and pathways. A growing number of studies have thus investigated microglial transcriptome remodeling over the course of neuropathological conditions and highlighted the molecular diversity of microglial response to different diseases. In the present work, we restrict our review to microglia obtained directly from *in vivo* samples and not cell culture, and to studies using whole-genome strategies. We first critically review the different methods developed to decipher microglia transcriptome. In particular, we compare advantages and drawbacks of flow cytometry and laser microdissection to isolate pure microglia population as well as identification of deregulated microglial genes obtained via RNA sequencing (RNA-Seq) vs. microarrays approaches. Second, we summarize insights obtained from microglia transcriptomes in traumatic brain and spinal cord injuries, pain and more chronic neurological conditions including Amyotrophic lateral sclerosis (ALS), Alzheimer disease (AD) and Multiple sclerosis (MS). Transcriptomic responses of microglia in other non-neurodegenerative CNS disorders such as gliomas and sepsis are also addressed. Third, we present a comparison of the most activated pathways in each neuropathological condition using Gene ontology (GO) classification and highlight the diversity of microglia response to insults focusing on their pro- and anti-inflammatory signatures. Finally, we discuss the potential of the latest technological advances, in particular, single cell RNA-Seq to unravel the individual microglial response diversity in neuropathological contexts.

## Introduction

Initially described almost 100 years ago by Pio Del Rio Hortega (Sierra et al., 2016), microglia are the resident immuno-competent cells of the central nervous system (CNS). They are in the first line for sensing and responding to any homeostatic changes in the CNS parenchyma (Ransohoff and Perry, 2009; Kettenmann et al., 2011, 2013). Compared to astrocytes and oligodendrocytes, microglia indeed rapidly react and can be considered as the chameleon within CNS glial populations. In addition to their immune-related functions, microglia are highly differentiated cells and actively participate in CNS wiring and modulation of neuronal activities (Tremblay et al., 2010; Schafer et al., 2012). Under physiological conditions, microglia are generally found in ramified/homeostatic state (previously referred as “resting”) in which they uninterruptedly screen their surrounding via their fine processes (Kettenmann et al., 2011). Microglia respond to any perturbation in CNS homeostasis ranging from acute trauma, normal aging and multiple neurodegenerative diseases. Activation process in microglia was traditionally defined by a gradual transformation from ramified into amoeboid morphology. However, this view has been recently challenged and it is now accepted that morphological alteration/activation does not necessarily reflect microglia function (or dysfunction; Perry, 2010; Ransohoff, 2016). In many neuropathological conditions, microglia are thought to have both positive and detrimental influences on disease progression (reviewed in Kabba et al., 2017).

Recent advances in cell-specific transcriptome profiling have been instrumental in uncovering microglial role in both physiological (Bédard et al., 2007; Gautier et al., 2012; Parakalan et al., 2012; Beutner et al., 2013; Butovsky et al., 2014; Orre et al., 2014a; Zhang et al., 2014; Solga et al., 2015; Bennett et al., 2016; Grabert et al., 2016; Matcovitch-Natan et al., 2016) and pathological conditions such as acute CNS traumatisms as well as in numerous chronic neurodegenerative diseases (Olah et al., 2012; Chiu et al., 2013; Hickman et al., 2013; Noristani et al., 2015, 2017). Several approaches have been used to isolate pure microglial population including flow cytometry (fluorescence-activated cell sorting, FACS) and laser microdissection. In addition, transcriptomic analyses have been carried out using microarrays and RNA sequencing (RNA-Seq).

In the current review, we first highlight the advantages and weaknesses of different approaches used to isolate pure microglial populations and discuss differences in transcriptome profiling using microarrays and RNA-Seq. Subsequently, restricting our review to: (1) microglia obtained directly from *in vivo* samples without further *in vitro* steps; and to (2) studies using whole-genome strategies, we summarize recent transcriptomic studies of microglia after traumatic brain and spinal cord injuries, pain and more chronic neurological conditions including amyotrophic lateral sclerosis (ALS), Alzheimer disease (AD) and multiple sclerosis (MS). Transcriptome profiling of microglia in other non-neurodegenerative CNS disorders such as peripheral immune challenges and gliomas are also presented. Finally, using a gene ontology (GO)-based classification, we present a comparison of the most activated pathways in each disease and highlight the diversity of microglia response to insults particularly focusing on their pro- and anti-inflammatory signatures. Lastly, we discuss the potential of the latest technological developments such as single cell RNA-Seq to unravel the individual microglial response diversity within different neuropathological contexts.

## Methods to Assess The Specific Microglial Transcriptome

The initial step towards cell-specific transcriptomic studies relies on obtaining sufficient cells of interest with the highest purity. As other immune cells of the CNS, microglia express a variety of cell surface molecules that can be used for purification through FACS. Until recently, no specific microglial cell surface marker was known thus microglia identification relied on the combination of different cell surface markers. Therefore, in many experiments designed to study the repertoire of genes expressed by microglia under physiological and/or pathological conditions, these cells have been identified based on CD11b positive and CD45 intermediate/low (CD11b^+^/CD45^low^) expression (Table 1). Such gating strategy not only allows discriminating microglia from other cells in the CNS, but also from infiltrating monocytes, as the latest are identified as CD11b^+^/CD45^high^ cells. However, it should be noted that CD45 expression levels can increase under pathological conditions, which may impair reliable separation between these two cell populations (Noristani et al., 2017). To overcome this issue additional cell surface markers including LY6C, CCR2 and CD44 can be combined with CD11b and/or CD45 to discriminate between microglia and infiltrating monocytes. Indeed, those markers are highly expressed by infiltrating monocytes but barely if not by microglia (Lewis et al., 2014). Few studies relied on a single cell surface marker, using either CD11b coated magnetic beads (Szulzewsky et al., 2015, 2016; Grabert et al., 2016), CD11b antibody (Noristani et al., 2015) or CD45 immunopanning (Zhang et al., 2014) to isolate microglia from CNS tissues. Although contamination by monocytes/macrophages may be negligible under physiological conditions, this is most likely not the case under pathological conditions in which specific contribution of microglia vs. infiltrating monocytes could not be resolved.

**Table 1:** Use of microglia-specific gene expression strategies in healthy and neuropathological conditions.

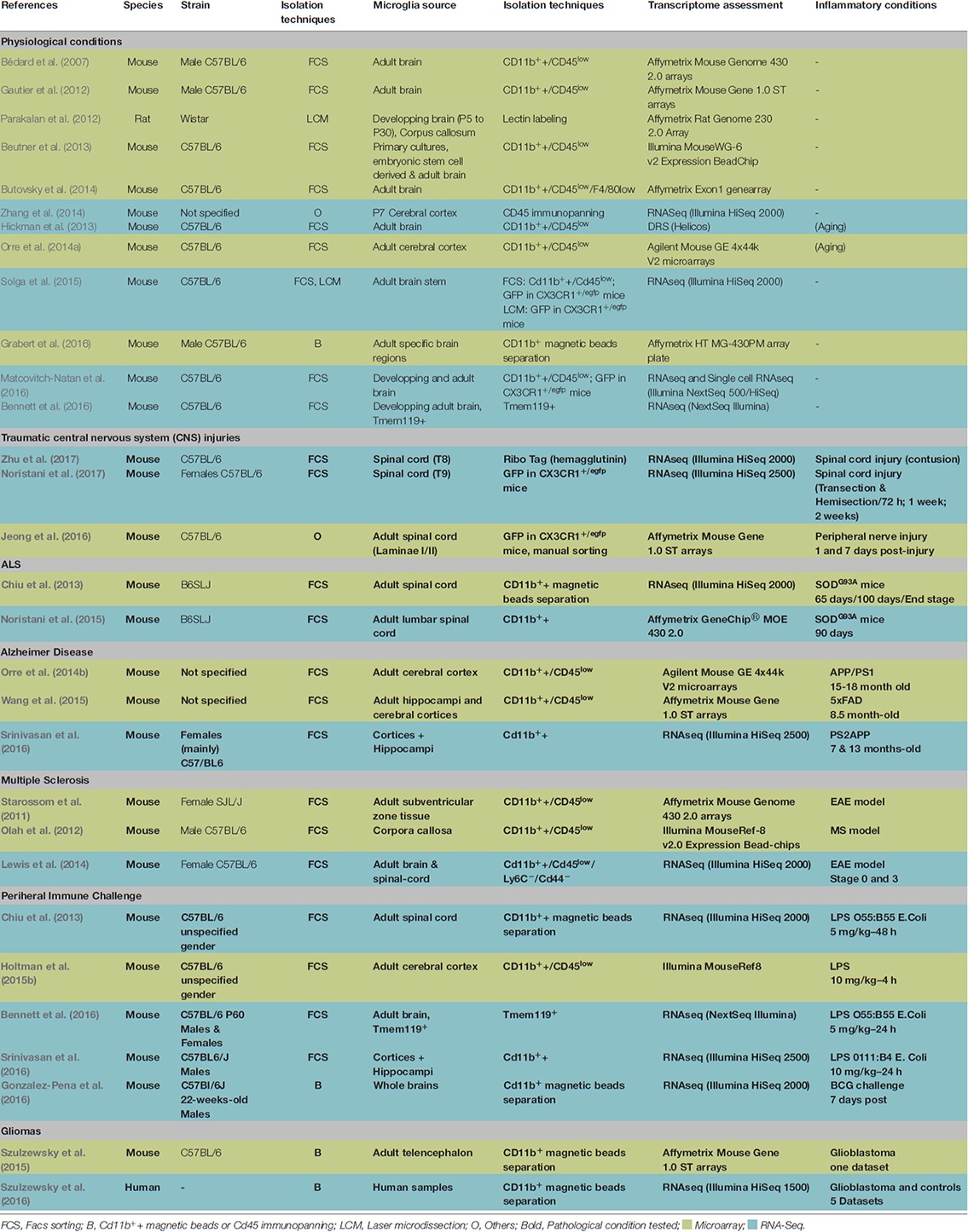

Interestingly Bennett et al. (2016) recently identified Transmembrane Protein 119 (TMEM119) as a microglia-specific cell surface marker and designed an antibody raised against the extracellular part of the protein to isolate pure microglia from brain tissue using FACS. The advantages of using this marker are that: (1) it is highly specific to microglia compared to other myeloid cells (including infiltrating monocytes); and (2) its expression level is claimed to remain constant under inflammatory conditions (however recent data suggest that it may not always be the case, see Keren-Shaul et al., 2017). However, TMEM119 expression is low in developing microglia and does not identify all microglia population before postnatal day 14 (P14; Bennett et al., 2016).

Several mouse lines with fluorescent markers of myeloid cells have also been generated, the most popular being the chemokine (C-X3-C motif) receptor 1 knock-in mice (CX3CR1^+/eGFP^; Jung et al., 2000). Three studies have taken advantage of this mouse line to study microglial transcriptome using either FACS (Solga et al., 2015; Matcovitch-Natan et al., 2016; Noristani et al., 2017) or laser capture microdissection (LCM) approaches (Solga et al., 2015). The main advantage of using such mouse line is that cells can be visualized or sorted without the need for antibody binding, a process that is time consuming and might interfere with microglia biology. Moreover, by restricting the gating to GFP^high^ cells, it is possible to almost exclusively select microglia and do not isolate potentially infiltrating inflammatory monocytes (Solga et al., 2015). However, the presence of contaminating anti-inflammatory monocytes could not be excluded, especially in case of traumatisms when the blood-brain-barrier and/or blood-spinal-barrier are compromised (Noristani et al., 2017). One potential drawback on this mouse line is that some studies reported behavioral differences compared to wild type (WT) littermate (Lee et al., 2010; Rogers et al., 2011). However, others revealed no microglial phenotype in CX3CR1^+/GFP^ compared to Iba1-EGFP mice (Hirasawa et al., 2005; Wolf et al., 2013). Additionally Solga et al. (2015) revealed that less than 0.5% of the detected genes differed between FACS isolated microglia from WT and CX3CR1^+/GFP^ microglia. Finally, another study combined lectin staining and LCM to isolate microglial cells from WT rat brainstem (Parakalan et al., 2012).

Isolating microglia through FACS or immunological-based procedures is a common approach that can lead to the isolation of several thousand cells at once. However, it requires tissue homogenization as a first step. This is generally achieved through mechanical, combined or not with enzymatic dissociation, two procedures that are likely to influence microglia biology. The whole isolation procedure takes between 3 and 5 hours and may involve the use of antibody binding (see above), which again might have further impact on microglial transcriptome. This being said, in their study (Bennett et al., 2016) have paid specific attention to this latter point and have shown that using their specific approach, the level of expression of genes known to be up-regulated upon microglia activation, such as *Il1β*, *Tnfα* and *Nfkb2*, was much lower in microglia isolated from naïve mice compared to lipopolysaccharide (LPS)-treated mice. This suggests that the transcriptome obtained from FACS isolated microglia is close to that of homeostatic microglia. The great advantage of LCM is that the procedure is likely to have lower impact on the microglia biology as the structure of the tissue is preserved until freezing of the CNS. Moreover, using LCM a much higher spatial resolution can be achieved for isolation of specific microglia subpopulations as it is feasible to isolate microglia from very close CNS regions/sub-regions. However, LCM suffers from two main disadvantages; first it is time consuming and only few hundred cells can be harvested at a time and second only partial enrichment in microglia cells can be achieved (i.e., generally between 5 and 10 times) as contaminants from surrounding cells cannot be excluded.

The second step in assessing microglial transcriptome is to measure gene expression after RNA extraction. Initial microglial transcriptome experiments were performed using DNA microarrays covering the entire genome, but recent development of NextGen sequencing (NGS) technology has provided a new path for gene expression analysis. Describing the intrinsic differences between the two techniques is beyond the scope of the present review, however in the next few lines we highlight the main differences of the two approaches. Both methods had been proved to be highly reproducible, though the main advantages of RNA-Seq over microarrays is the higher dynamic range, which allows for the detection of more differentially expressed genes with greater fold-change (Wang et al., 2014). Importantly, RNA-Seq is more sensitive and can therefore detect low abundance transcripts (Bottomly et al., 2011). These features, i.e., increased sensitivity and high dynamic range, are crucial when assessing differential gene expression between physiological and pathological conditions, especially in immune cells in which dramatic induction or repression of gene expression is expected. In addition, because RNA-Seq does not rely on pre-designed probes, it is devoid of issues associated with probe redundancy and annotation. Finally, RNA-Seq is superior in detecting different biologically critical isoforms. However, although costs are dropping with introduction of new sequencing systems, RNA-Seq is still more costly than microarrays (Yandell, 2015). Additionally, while methods for analyzing microarray data are fully mature and straightforward, there is no consensus on which pipelines to use when analyzing RNA-Seq data (Zhang et al., 2014; Huang et al., 2015).

## Transcriptome Profiling of Microglia in Neurodegenerative Conditions

As pointed in the previous section, depending on the strategy employed for microglia isolation, other immune cells particularly inflammatory monocytes, can contaminate the preparation. In the following sections, we refer to microglia when the strategy employed is unlikely to give rise to contamination by infiltrating monocytes, and to microglia/monocytes or microglia/macrophages when peripheral immune cell contamination cannot be excluded.

### Traumatic Central Nervous System Injuries

CNS response to traumatism is a complex phenomenon involving numerous structural, biochemical and transcriptomic alterations. CNS traumatisms not only trigger reactivity in resident microglia, they also disrupt the blood-brain-barrier and/or blood-spinal-barrier, which lead to the infiltration of peripheral monocytes. Altogether microglia and infiltrating monocytes drive inflammation after CNS injuries. In the following sections, we will discuss the role of microglia/monocytes in traumatic brain injury (TBI) and spinal cord injury (SCI).

#### Traumatic Brain Injury

TBI is an overwhelming health problem with annual incidents of 295 per 100,000 inhabitants worldwide (Nguyen et al., 2016). Given that majority of TBI sufferers ranges between 15–24 years of age, the social/economic cost associated with TBI can reach over $4 million per case (Dash et al., 2004). Clinical symptoms linked to TBI include impairments of motor function, cognition, memory, attention and motivation that depend on anatomical location and traumatism severity. Currently, there is not effective treatment to minimize disabilities due to TBI.

TBI triggers a marked inflammation driven by resident microglia and infiltrating peripheral monocytes that play a fundamental role in subsequent regeneration and plasticity (Hernandez-Ontiveros et al., 2013). Microarray studies had focused on genomic changes in different brain regions after TBI (Kobori et al., 2002; Matzilevich et al., 2002) and subsequent approaches, using both microarray and quantitative real time PCR (qRT-PCR) of the whole brain at several time-points following injury (1, 4, 12 and 24 h as well as 3 and 7 days), showed upregulation of several inflammatory cytokines transcripts including *Ccl2*, * Cxcl2*, *Il6* and *Tgf-βI* (Wei et al., 2009). More recently Cao et al. (2012), using qRT-PCR, demonstrated predominant upregulations of inflammatory and neurotoxic transcripts such as *MhcI*, *MhcII, Tspo*, *Tnfα*, *Cd45*, *TgfβI* and *TgfβRII* at 7 and 28 days after TBI.

Although inflammation and up-regulation of inflammatory cytokines is predominant in brain transcriptomic alterations after traumatism (Redell et al., 2013; Samal et al., 2015), to our knowledge, no cell-specific study has been carried out so far to examine gene profile modifications specifically in microglia/monocytes following TBI. There is thus a clear need for such analysis. In addition, these studies should not only focus on microglia/monocyte gene alterations at multiple stages after different injury severities, but also need to take into account their proximity to the lesion site and different brain regions. These studies will be instrumental in deciphering the respective roles of microglia and monocytes following TBI and will uncover novel cell-specific therapeutic approaches for minimizing TBI-associated disabilities.

#### Spinal Cord Injury

SCI is a traumatic event with dire consequences on the physical and emotional welfare of affected individual. There are between 8 and 246 annual cases of SCI per 100,000 individuals worldwide, which induce high socio/economic costs to our society (Furlan et al., 2013). Clinical symptoms linked to SCI depend on the anatomical level and the severity of the injury ranging from minor sensory/motor weakening to complete quadriplegia.

Microglia, are not only the first responsive glial cells after SCI (Tian et al., 2007), but they also participate in the recruitment of peripheral monocytes to the injury site (David and Kroner, 2011). Reports have shown both pro- and anti-regenerative roles of microglia and monocytes after SCI (David and Kroner, 2011). Phagocytosis of cellular debris (Perrin et al., 2005b) and expression of neurotrophic factors (Lambertsen et al., 2009) are examples of beneficial effects of microglia and monocytes after SCI (Mukaino et al., 2010).

An earlier report using microarray and qPCR analyses of the whole spinal cord reported a predominant over-expression of neurotoxic genes at 1, 3, 7, 14 and 28 days following SCI (Kigerl et al., 2009). More recently Kroner et al. (2014), using flow cytometry, also showed that microglia/macrophages mostly over-express neurotoxic factors at 1, 4 and 15 days after SCI. However, this latter report had focused on a relatively small number of selected transcripts as opposed to cell-specific genome-wide analyses. Using RNA-Seq on the whole rat spinal cord at 1, 6 and 28 days following contusive injury Shi et al. (2017) recently identified that the most enriched pathways include “immune response”, “MHC protein complex”, “antigen processing and presentation”, “constituent of ribosome”, “ion gated channel activity”, “small GTPase-mediated signal transduction”, “cytokine and/or chemokine activity and signaling”, “axon guidance” and synaptic (dopaminergic, glutamatergic and GABAergic) transmission”. In a recent study Zhu et al. (2017) combined RiboTag method to isolate infiltrating macrophage-specific RNA from the spinal cord of mice that underwent contusive injury and RNA-Seq to obtain the transcriptomic profile at 3 and 7 days post-injury. The transcriptomic profile of macrophages at 3 days post-injury revealed an enrichment of genes involved in diverse processes including “migratory behavior”, “cell and biological adhesion”, “taxis and chemotaxis”, “cytokine-cytokine receptor interaction” and “chemokine signaling pathways”. In contrast, at 7 days post-injury, enriched biological processes related almost exclusively to “lipid catabolism” comprising glycolipid, glycosphingolipid and sphingolipid catabolism. Five molecules associated with lipid catabolism were identified as network hubs (*Tnf* being decreased and *Cd36*, *Lpl*, *Pparγ* and *Abca1* increased; Zhu et al., 2017). Recently, we published an extensive SCI-induced transcriptomic analyses of microglia/macrophages at multiple stages after different lesion severities (Noristani et al., 2017). We used the CX3CR1^+/eGFP^ mice to isolate a microglia/macrophages population by flow cytometry. Comparing lateral hemisection and complete section of the spinal cord, as moderate and severe injury models, we investigated microglia/macrophages transcriptomic responses at 3, 7 and 14 days post-injury using RNA-Seq and pathway analyses. In contrast to astrocyte (Noristani et al., 2016, 2017), microglia/macrophages responses after injury are time- but not severity-dependent. Using pathway analyses, we identified that at 3 days post-injury, microglia/macrophages responses largely involve proliferation, whilst at 7 and 14 days they regulate “inflammation, defense response”, “cytoskeleton” and “extracellular matrix remodeling” (Supplementary Table S1). Moreover, after both moderate and severe SCI, microglia/macrophages displayed a dual transcriptomic phenotype with an earlier increase in potentially neuroprotective genes followed by a concomitant over-expression of possibly neurotoxic and neuroprotective transcripts. Microglia/macrophages-specific transcriptomic analysis also permitted to identify that SCI induces the expression of astrocytic markers at mRNA and protein levels such as glial fibrillary acidic protein (GFAP) and vimentin (VIM) in microglia as early as 3 days post-injury that persisted up to 6 weeks post-traumatism (Noristani et al., 2017). These data raise awareness on the specificity of accepted glial markers when studying pathological conditions. The potential role of SCI-induction of astrocytic markers in microglia is currently unknown but demonstrates novel insights into microglia plasticity. Moreover, pathway analysis highlighted the putative involvement of DNA damage and in particular *Brca1* in microglia/macrophages, thereby broadening our previous findings in microglia/macrophages from ALS mouse model and patients (Noristani et al., 2015). These findings suggest the involvement of oncogenic proteins in microglia after CNS insults. Future studies aimed at manipulating these oncogenic proteins in microglia are necessary to uncover their roles in CNS pathologies.

### Chronic Neurodegenerative Conditions

#### Amyotrophic Lateral Sclerosis

ALS is a rare neurodegenerative disease with a prevalence that ranges between 1 and 6 per 100,000 per year. ALS is characterized by a selective and progressive degeneration of upper and lower motoneurons that induces progressive muscle atrophy and paralysis. Majority of patients die within 3–5 years mainly due to respiratory failure. Approximately 10% of ALS cases are familial with identified genetic mutations whilst 90% of ALS patients are sporadic cases (Leblond et al., 2014). Growing evidences indicate that motoneuron death results from a combination of cell autonomous dysfunctions (intrinsic neuronal deregulation) and non-cell autonomous contributions from neighboring cells (reviewed in Lee et al., 2016). In particular, microglia actively participate in ALS pathogenesis through their orchestration of the inflammatory response (reviewed in Bowerman et al., 2013). Amongst animal models of ALS, transgenic mice carrying a human mutated form of the super oxide dismutase 1 (SOD1) gene were the first engineered and are still the most widely used (reviewed in Picher-Martel et al., 2016). Studies using postmortem tissues and animal models enlighten microglia involvement in ALS pathogenic cascade (Gerber et al., 2012), pinpointing to their dual protective and detrimental roles over the course of the disease (reviewed in Brites and Vaz, 2014; Philips and Rothstein, 2014).

Microglia/macrophages-specific gene profiling in SOD1^G93A^ had been carried out in two recent studies. In the first study, using whole spinal cords of B6/SJL SOD1^G93A^ transgenic mice, Chiu et al. (2013) analyzed microglial transcriptome at three time points corresponding to characteristic phases of disease development: i.e., day 65 (pre-symptomatic phase), 100 (intermediate phase) and 130 (end stage). In fact, 65 days of age better corresponds to an early symptomatic phase as demonstrated in a previous study (Gerber et al., 2012). Microglia were isolated using CD11b^+^ magnetic beads (see Table 1). First, the authors demonstrated that SOD1^G93A^ microglia were not derived or contaminated by infiltrating monocytes, as there were very few CD11b^+^, CD45^+^ and Ly6C^+^ cells in their isolated population. Second, *Olfml3*, *Tmem119* and *Siglec-H* mRNA expressions were shown to increase in a progressive age-dependent manner. Furthermore, an overall significant increase of the 29 microglia specific markers previously identified in the study was observed in SOD1^G93A^ microglia. Third, SOD1^G93A^ microglia displayed transcriptional profile presenting a concomitant deregulation in genes that may play either neuroprotective or neurotoxic effects. A significant upregulation of osteopontin (*Spp1*) was observed; this secreted factor had been shown to play a neurotoxic role in encephalomyelitis and AD; whilst having a neuroprotective role after SCI (Hashimoto et al., 2007). De-regulated genes that may play a neurotoxic role include matrix metalloproteinase 12 (*Mmp12)*, optineurin (*Optn*), tumor necrosis factor α (*Tnfα*), *Il1α*, *Il1β*, receptors for type 1 IFNs (*Ifnar1* and *Ifnar2*) and IFN response genes (*Ifit1*, *Ifit3*, *Ifitm3* and *Igip30*), receptor for IL-10 (*Il-10ra*) and Nox2 (*Cybb*). Concomitantly, deregulation of potentially neuroprotective genes such as *Igf1*, *progranulin*, triggering receptor expressed on myeloid cells 2 (*Trem2*) and its downstream adaptor molecule *Dap12* (*Tyrobp*) were also observed in SOD1^G93A^ microglia. Furthermore, lysosomal pathways (including cathepsins and genes related to AD such as Tau (*Mapt*), Presenilin 2 (*Psen2*) and Apolipoprotein E (*ApoE*) were enriched in SOD1^G93A^ microglia. The authors hypothesized that these pathways may have implication in protein clearance and neurodegeneration (Chiu et al., 2013).

In the second study, using lumbar segment of B6/SJL SOD1^G93A^ spinal cords, Noristani et al. (2015) analyzed the microglia/macrophages transcriptome of 90 days old mice (symptomatic phase) and compared microglia/macrophages molecular signature to that of motoneurons previously obtained by the group using the same strain of mice (Perrin et al., 2005a). Microglia/macrophages were isolated using CD11b^+^ antibody and transcriptome profiling was obtained by microarrays (see Table 1). GO analysis, allowed identifying deregulations in “chemotaxis”, “angiogenesis”, and “inflammation networks” (Supplementary Table S1). When analyzing cellular processes in SOD1^G93A^ microglia/macrophages, the immune response process was the most modified, in particular through a down-regulation of the gene coding for alpha-synuclein and an up-regulation of *Ccl5* and *Cxcl13* transcripts. In contrast to the previous study (Chiu et al., 2013), a concomitant continuum, rather than a sequential expression, of neuroprotective and neurotoxic states was observed, presenting both neuroprotective (up-regulation of *Clec7a*, *Igf1*, *Mmp12*, *Spp1* and *Lgals3* and down-regulation of *Retnla* and *F13a1*) and a neurotoxic phenotype (up-regulation of *Cd86*, *Tnfα*, *Bcl2a1a* and *Cxcl10* and down-regulation of *Gadd45gip1*). In addition, we identified the deregulation of genes involved in “blood coagulation” and “hypoxia”. Interestingly, microglia/macrophages transcriptomic profile highlighted the altered expression of several genes pointing toward the tumor suppressor breast cancer susceptibility gene 1 (*Brca1*). Comparison with our previous data on microdissected motoneurons (Perrin et al., 2005a) from the equivalent lumbar segment of B6SJL-Tg-SOD1^G93A^ spinal cords substantiated the putative contribution of *Brca1* in ALS, since both in microglia/macrophages and motoneurons pathway analysis pointed toward *Brca1*. The relevance of this finding was further enlightened by the finding that BRCA1 protein is specifically expressed in human spinal microglia and is up-regulated in ALS patients (Noristani et al., 2015).

**Figure 1:**
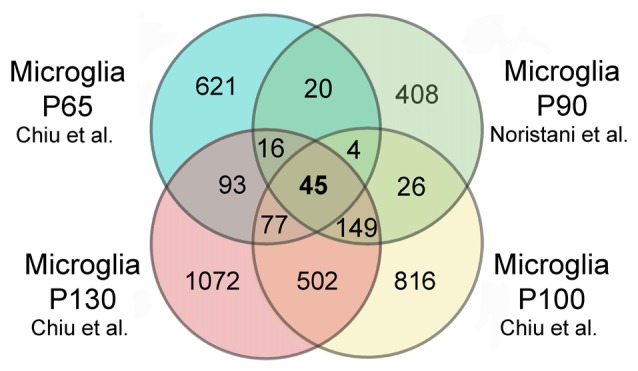
*In silico* comparison of gene dysregulation in super oxide dismutase 1 (SOD1)^G93A^ microglia. Data compared are from the original publications of Chiu et al. (2013) and Noristani et al. (2015). Venn diagrams showing that 45 genes are commonly dysregulated in SOD1^G93A^ microglia at all time points in both studies, corresponding to 0.9% of the dysregulated genes. *Brca1* is amongst these 45 genes. A total of 5117 genes were identified as dysregulated, 621 genes are uniquely dysregulated in hSOD1^G93A^ microglia at P65, 408 at P90, 816 at P100, and 1072 at P130. 1.17% of the identified genes are commonly dysregulated at P65 and P90; 4.4% are commonly modified at P90 and P100 and, 15% are commonly dysregulated at P100 and P130. In the study of Chiu et al. (2013) microglia were isolated by FACS from the whole SOD1^G93A^ spinal cord and RNA sequencing (RNA-Seq) was used. In the study of Noristani et al. (2015) microglia were isolated by FACS from the lumbar segment of the SOD1^G93A^ spinal cord and microarrays were used. P65 correspond to onset/early symptomatic phase, P90 early symptomatic, P100 symptomatic and P130 the end stage of the disease. Thresholds to select the genes were identical in the two studies with a fold change >2 and *p* value (FDR) <0.05.

Interestingly, in *silico* comparison of the two studies (Chiu et al., 2013; Noristani et al., 2015) revealed 45 commonly de-regulated genes at all time points in both studies (Figure 1). However, altered expression of genes such as *Il1α*, *Ilβ*, *Il-10*, *Ifnar1* and *Ifnar2* as well as *Nox2* observed in Chiu et al. (2013) study were not confirmed in our study (Noristani et al., 2015). These discrepancies, may result from the segment of the spinal cord that had been analyzed (whole vs. lumbar) or the method used to assess the microglial transcriptome (RNA-Seq vs. microarrays).

#### Alzheimer’s Disease

Dementia affects over 35 million people worldwide (Rizzi et al., 2014), with Alzheimer’s disease (AD) representing about 60% of the total cases (Qiu et al., 2009). AD is a chronic neurodegenerative disorder characterized by progressive and irreversible memory loss, as well as impaired cognitive functions. Histopathological hallmarks of the disease are the presence of extracellular aggregated amyloid-β deposits (Aβ plaques) and intra-neuronal aggregates of hyper-phosphorylated tau (Selkoe, 2001). Plaques are surrounded by reactive microglia and astrocytes (Itagaki et al., 1989). The initial thought was that microglia aggregation around plaques and their transition into an active/amoeboid phenotype was triggered by Aβ plaques deposition (Kamphuis et al., 2012; Orre et al., 2013). However, the role of microglia in the AD neuropathology still remains unclear, and several studies have shown that some microglia functions (i.e., phagocytosis and TGFβ secretion) may be beneficial, whereas others may be deleterious (i.e., secretion of pro-inflammatory cytokines), or may even lack major impact on the pathology (reviewed in Morgan et al., 2005; Aguzzi et al., 2013). Interestingly, genome wide association study (GWAS) analyses of sporadic AD cases have shown that variants of highly expressed microglial transcripts (including: membrane-spanning 4-domains sub family A member 6A (*Ms4a6a*), *Cd33* and *Trem2*, are associated with an increased risk of AD, thus suggesting that microglia play an important role not only in the development but also in the onset of the disease (Heneka et al., 2014; Karch and Goate, 2015). In addition, a recent study suggests that AD-associated genetic susceptibility mainly affects microglia (Skene and Grant, 2016).

So far, the involvement of microglia in Aβ pathology has primarily been investigated through the use of *in vitro* culture approaches and/or immunostainnings of mouse or human AD tissues. Transcriptome studies in relation to AD have been mainly based on the RNA extracted from whole tissue (Bossers et al., 2010; Wirz et al., 2013; Zhang et al., 2013). However, three recent studies reported specific microglia transcriptomic analyses in different transgenic mouse models of AD, and at different stages of the pathology (Orre et al., 2014b; Wang et al., 2015; Srinivasan et al., 2016; see Table 1 for details). Two of them were performed using microarrays (Orre et al., 2014b; Wang et al., 2015) whereas Srinivasan et al. (2016) employed an RNA-Seq approach.

The study of Wang et al. (2015) was not aimed at studying in details the remodeling of the microglia transcriptome in the context of AD. Although the authors reported a profound transcriptomic remodeling in microglia isolated from 8.5 months old 5xFAD transgenic mice, which represents a late stage of the disease in this model, they mainly describe an increase in the expression of transcripts associated with microglial activation (*Mhc-II*, *Cd11c*), production of inflammatory cytokines (*Tnfα*, *Il12*, *Spp1*) and neurotrophic factors (*Igf1* and *Vegfa*).

More recently (Srinivasan et al., 2016) performed RNA-Seq analyses of the repertoire of genes expressed in microglia/monocytes isolated from PS2APP transgenic mice at 7 and 13 months of age, as representatives of intermediate and late stages of the disease, respectively. The authors identified 249 differentially expressed genes in microglia/monocytes purified from PS2APP transgenic vs. non-transgenic mice at 13 months of age. An interesting finding, is that the deregulated genes pointed towards an altered lipoprotein metabolism in microglia from plaque-ridden tissue. They concluded that such microglial dysfunction may contribute to the altered processing of the amyloid precursor protein (APP) and Aβ deposition.

So far, the most detailed analyses of transcriptomic changes observed in microglia in a model of AD arise from the study of Orre et al. (2014a) performed in CD11b^+^/CD45^int^ sorted microglia from 15 to 18 months old APPswe/PS1dE9 (APP/PS1) transgenic mice (for details see Table 1). In this late phase of the disease, they identified 1119 deregulated genes in microglia from transgenic mice compared to age-matched WT mice (fold change > 1.5, corrected *p*-value < 0.05), about 25% of which are highly enriched in microglia. The top GO classes associated to the upregulated genes are “cytokine activity”, “defense response”, “T-cell activation”, “cholesterol metabolic processes” and “regulation of programmed cell death” (Supplementary Table S1), whereas those associated with the down-regulated genes were “carbohydrate binding”, “nucleoside tri phosphatase regulator activity” and “endocytosis”, but also “immune response” and “cytokine activity”. By comparing, at the same time, the transcriptomic remodeling in both microglia and astrocytes, the authors revealed that microglia are most likely the main contributors to the levels of pro-inflammatory genes in AD whilst astrocytes may contribute to the maintenance of microglia in an activated state.

Remarkably, Orre et al. (2014a) and Srinivasan et al. (2016) identified significant deregulation in genes involved in lipoprotein metabolism in microglia purified from late stage brains of two independent transgenic mouse models of AD. Specifically, *ApoE* and *Ldlr* genes have been shown to be important for brain-to-blood Aβ clearance (Castellano et al., 2012). *ApoE* and *Ldlr* are among the most deregulated microglial genes in AD, which suggest that impaired lipoprotein metabolism in microglia may play a key role in the late phase of the disease.

In addition to their interesting findings, these studies revealed the interest in studying cell-specific transcriptomic remodeling in AD. Further studies are however needed to better understand the role of microglia in this neuropathology. The cited studies mainly focused on late stage of the disease, however, it is critical to understand the role of these cells in the early, pre-symptomatic, stages of the disease. Indeed, given their ability to respond to trivial changes in CNS parenchyma, microglial transcriptomic alterations at early stages of the disease may provide valuable biomarkers for early AD detection.

So far, microglia specific transcriptomic studies have been performed based on Aβ pathology in animal models of AD. Whereas in humans, brain regions that are particularly relevant to AD such as the hippocampus, present low plaque loads and high levels of hyper-phosphorylated tau aggregates, in animal models the presence of intra-neuronal aggregates is not observed. Interestingly, a recent qPCR-based study reported a degenerative profile for microglia in human AD hippocampi, which may account for the cognitive deficits observed (Sanchez-Mejias et al., 2016). The authors proposed that the toxic effect may be mediated through phagocytosis of neurons containing intracellular phospho-tau. This evidence suggests that characterization of microglia responses should be performed in different animal models of the pathology and that results should be confronted to get a better knowledge of the involvement of microglia in AD.

In addition, immunohistological studies in both animal models and in humans have highlighted that there are at least two distinct microglia sub-populations in AD: the activated/amoeboid microglia that cluster around the amyloid plaques and the resting/ramified microglia disseminated away from the plaques (Ruan et al., 2009; Serrano-Pozo et al., 2016). Determining the respective roles of these two populations may help designing better targeted therapeutic strategies by preventing the deleterious effects of microglia while boosting their beneficial roles in AD neuropathology.

During the final review process of the present manuscript, a remarkable and extensive study, which tackles some of the questions raised above, was published (Keren-Shaul et al., 2017). Indeed, using a single-cell RNA-Seq approach, these authors have mapped all immune cell populations present in WT and 5xFAD transgenic mice and have identified a new subtype of microglia, which they refer to as disease associated microglia (DAM). Interestingly, DAM are more abundant in regions that display high levels of amyloid plaque burden. DAM activation occurs in two sequential steps: the initial step is Trem2-independent and is associated with the downregulation of microglia homeostatic markers such as P2ry12. The second step is Trem2-dependent and is characterized by over-expression of transcripts involved in lipid metabolism pathways and in phagocytosis related functions. Thus, the authors proposed that DAM are needed to mitigate the disease through phagocytosis but that this protective function occurs only in the late phase of the disease. Importantly, DAM were also identified in AD patient (Keren-Shaul et al., 2017).

#### Multiple Sclerosis

MS is an autoimmune inflammatory demyelinating disorder affecting the CNS characterized by inflammation, gliosis and axonal injury. Onset of the disease is observed between 20–40 years of age and the progressive disease phase occurs between 5–35 years following the disease onset. Symptoms that are manifested episodically and partly reversible may ultimately lead to loss of mobility (reviewed in Ransohoff et al., 2015). Phagocytic microglia are observed when demyelination occurs in the CNS and they actively participate in myelin debris clearance. When clearance is blocked, proper remyelination is impaired attesting to the positive role of microglia in demyelinating disorders such as MS. However, microglia also play a detrimental role in MS through production of inflammatory molecules (see review in ElAli and Rivest, 2016). A recent study of the expression profile of genes involved in inflammation, show that conversely to microglia, monocyte-derived macrophages are highly phagocytic and inflammatory, whereas microglia metabolism was robustly down-regulated (Yamasaki et al., 2014).

Using a model of experimental autoimmune encephalomyelitis (EAE) that recapitulates some aspects of MS, Starossom et al. (2011) isolated microglia from the sub ventricular zone at several phases of the disease including acute EAE (around 13 days post-induction) and chronic EAE (after the first relapse and corresponding to between 50 to 60 days post-induction). A combination of FACS and microarray analysis was used to reveal both common and distinct microglial transcriptomic profiles in acute and chronic EAE. GO ranking identified as enriched categories predominantly during the acute phase “cellular growth and proliferation”, “cellular movement” and “cell-to-cell signaling”. Genes related to “immunological disease”, “connective tissue disease” and “inflammatory response” were enriched during both phases of EAE (Supplementary Table S1). In contrast, during chronic EAE genes associated with reelin signaling, ephrin receptor signaling and phospholipase C signaling were enriched. Further functional network analysis highlighted that the up-regulation of niche supporting factors observed during the acute EAE phase may reflect a dual beneficial and detrimental role of microglia on neural stem cells (Starossom et al., 2011).

Subsequently, to better understand the role of microglia in remyelination processes, gene expression profile of microglia from the corpus callosum of mice presenting primary cuprizone-induced demyelination was performed (Olah et al., 2012). This mouse model mimics some aspects of MS since following primary toxin-induced demyelination a spontaneous remyelination is observed. Microglia were obtained using CD11b^+^/CD45^low^ flow cytometry selection and genome-wide data were acquired by means of microarrays (see Table 1 for details). Specific deregulated genes were identified in microglia during demyelination (cuprizone diet for 5 weeks) and remyelination (cuprizone diet for 5 weeks followed by 2 weeks without cuprizone). However, gene profiles highlighted that the same microglia population underwent a gradual phenotype modification (from demyelination to remyelination) as opposed to distinct microglia populations associated with either demyelination or remyelination. Demyelination was characterized by deregulation of genes involved in phagocytosis (including *Lrp1*, *Calr*, *Cd14*, *Itgb2*, *Itgam* and *Lgals*3). Conversely, deregulation of genes involved in the recruitment of oligodendrocyte precursor cells and support for tissue remodeling (including *Mmp1*2, *Mmp14*, *Cxcl10*, *Cxcl1*3, *Igf1*, *Tgfb1*, *Pdgfa* and *Pdgfb*) characterized the remyelination phase.

In another study, Lewis et al. (2014) uncover the respective contribution of microglia and monocyte-derived macrophages over the course of EAE. They combined the use of several surface markers such as CD45, LY6C and CD44 to discriminate these two cell populations by flow cytometry and RNA-Seq on days 7 and 14 post-immunization (See Table 1). In both microglia and monocyte-derived macrophages activation markers such as *Cd86* and *Cd80* were up-regulated, however, many of these activation markers showed a higher up-regulation in monocyte-derived macrophage as compared to microglia. In particular, *Sell*, *Cd69* and *Cd40* were not up-regulated in microglia. Analysis at protein level confirmed the highest expression level of activation markers in monocyte-derived macrophages as compared to microglia. Interestingly, microglia did not express MHCII and CD80 upon activation. Also, the authors identified a lower proliferative capacity of microglia when compared to monocyte-derived macrophages as attested by the greater up-regulation of Ki67 in the latest cell population. Another major difference between the two cell populations, were the distinctive transcriptomic profiles of chemokines, phagocytosis genes, complement genes and transcription factor (reviewed by Crotti and Ransohoff, 2016). Genes involved in the process of phagocytosis and phagoptosis such as *Mertk* and its ligands *Pros1*, *C1q* component, C1q receptors (*Lrp1* and *Calr*) and *Gas6* were up-regulated in microglia over the course of EAE strongly suggesting the important role of microglia in clearance of apoptotic cells in myelin debris in MS (Lewis et al., 2014).

#### Pain

Pathological pain is one of the most important public medical issues, which seriously undermines the quality of life. Neuropathic pain is one of the most difficult forms of pain to treat (Dworkin et al., 2003; Costigan et al., 2009). It is a complex, chronic state usually accompanied by tissue injury with dysfunctional or injured nerve fibers. Clinical symptoms associated with neuropathic pain include abnormal sensations such as dysesthesia or pain from normally non-painful stimuli (allodynia). Between 3% and 8% of the population from industrialized countries are affected, and in 5% of the affected patients, it may be severe. The estimated community prevalence of neuropathic pain from the clinical examination is 9.8%. Neuropathic pain may result from disorders such as diabetes and cancer or physical damage such as road accidents and are challenging to treat due to the current lack of effective therapies controlling pain in patients. Microglia are recognized as important participants in mechanisms of sensory encoding and the plasticity underlying the generation of spinal sensitization, hyperalgesia and chronic pain (Tsuda, 2016). Indeed, in response to peripheral nerve injury, spinal microglia become reactive (Smith, 2010) and contribute to central sensitization, the key mechanism for the development of neuropathic pain.

To better understand how changes in spinal microglia affect neuron under developmental stage of neuropathic pain, Jeong et al. (2016) performed transcriptomic analysis of individually collected pools of low numbers of spinal microglia located in laminae I and II of the dorsal horn. Neuropathic pain was induced by transection of the L4 spinal nerve in CX3CR1^+/eGFP^ mice. Microglia were identified as GFP expressing cells and collected with glass pipette from superficial laminae I/II at L3–L5 spinal dorsal horn level. The transcriptomes of pools of 10 cells were identified using microarrays. Early and late microglial transcriptomic changes (compared to sham operated controls) were investigated at 1 and 7 days post-injury, respectively. A total of 559 deregulated genes were found after peripheral injury, with only 27 transcripts commonly deregulated at the two time-points. Accordingly, functional analysis of the deregulated genes revealed distinct transcriptomic profiles for microglia in the early and late phases of neuropathic pain development. This suggests that although early and late microglia subtypes cannot be distinguished by morphology, their functional phenotypes display considerable differences. In the early phase, genes related to the sensing functions of microglia dominate with “chemotaxis” and “complement signaling pathways” being the most deregulated biological function. In contrast, in the late phase, genes involved in signaling pathways were more prevalent with an over-representation of the “IL6 production”, “Chemokine biosynthetic process” and “JNK cascade” genes sets (Supplementary Table S1). Interestingly, the cellular component associated to the deregulated genes differs between the early and late phase of the pain development. The authors suggest that the expression profiles they unraveled may be responsible for the transition from initiation to maintenance of the neuropathic pain. Importantly, through temporal variation analysis this study also point to *miR-29c* and *Gria* as critical factors of microglia activation during mechanical allodynia.

## Transcriptome Profiling of Microglial in Non-Neurodegenerative Conditions

### Peripheral Immune Challenges

Infection by pathogenic microorganisms triggers an acute phase response that manifests itself with fever, neuro-endocrine and behavioral changes. The so called “sickness behavior” characterized by non-specific symptoms such as malaise, fatigue, anorexia, hypo- and hypersomnia, depression and lethargy is accompanying severe infections (Hart et al., 1988). In animal model of sepsis, administration of LPS, the active fragment of gram-negative bacteria, either peripherally or directly into the brain, induces cognitive impairments and behavioral disturbances reminiscent of the sickness behavior observed in humans (Reichenberg et al., 2001; Godbout et al., 2005; Krabbe et al., 2005). In parallel to the behavioral changes, sickness behavior has been also associated, in both rodents and humans, with increased neuroinflammation and more specifically increased microglia reactivity (Semmler et al., 2005; Lemstra et al., 2007). The molecular mechanisms connecting systemic inflammation, microglial reactivity and sickness behavior remain unclear but recent studies suggest that microglia reactivity play a pivotal role in the development of sickness behavior (Xu et al., 2015).

Few studies have reported microglia transcriptome changes after induction of systemic immune challenges (Table 2). Three studies were based on the acute effects of LPS administrations (Chiu et al., 2013; Bennett et al., 2016; Srinivasan et al., 2016), whereas Gonzalez-Pena et al. (2016) investigated the effect of Bacille Calmette Guérin (BCG) 7 days after the challenge. The main purpose of the first three LPS based studies (Chiu et al., 2013; Bennett et al., 2016; Srinivasan et al., 2016) was not to study the remodeling of the microglial transcriptome after LPS/sepsis but rather to compare: (1) the transcriptome of “classically” activated microglia (i.e., after LPS challenge) vs. microglia isolated under specific pathological/physiological conditions (i.e., in ALS context (Chiu et al., 2013) or; (2) during brain development (Bennett et al., 2016); or (3) to compare the transcriptomic changes observed in different brain cell populations (Srinivasan et al., 2016). Consequently, these studies did not report extensive characterization of the biological effects of the LPS-mediated immune challenge. Yet, Bennett et al. (2016) reported that 24 h after LPS administration, microglia upregulated Toll-like receptor, Vitamin D3 receptor/RXR activation and “acute phase signaling pathways”, whereas by 48 h following LPS challenge, spinal-cord microglia/monocytes were enriched in “DNA replication”, “cell cycle” and “innate immune signaling” through the RIG-I-like receptor and NOD-like receptor KEEG pathways (Supplementary Table S1; Chiu et al., 2013). Interestingly, both studies revealed that although these different microglia subtypes up-regulated several classes of genes associated with myeloid cell activation and displayed activated phenotypes, LPS-activated microglia displayed a distinct transcriptomic profiles as compared to microglia isolated from SOD1^G93A^ spinal cord (Chiu et al., 2013) or from E17 mouse brain (Bennett et al., 2016). On the other hand Srinivasan et al. (2016) revealed numerous additional LPS-responsive microglial/monocytes genes and although these authors did not perform any gene enrichment analysis they showed that LPS produced a robust and specific RNA processing response, including alteration of alternative splicing.

**Table 2:** Use of microglia-specific gene expression strategies after peripheral immune challenges.

References	Species	Microglia source	Isolation techniques	Isolation techniques	Transcriptome assessment	Immune challenge	
Chiu et al. (2013)	Mouse	Adult spinal cord	FCS	Cd11b^+^+ magnetic beads separation	RNAseq (Illumina)	LPS O55:B55 E.Coli (Sigma) 5 mg/kg; i.p.	48 h
Bennett et al. (2016)	Mouse	Adult brain, Tmem119+	FCS	Tmem119+	RNAseq (NextSeq Illumina)	LPS O55:B55 E.Coli (Sigma) 5 mg/kg; i.p.	24 h
Gonzalez-Pena et al. (2016)	Mouse	Whole brains	B	Cd11b^+^ magnetic beads separation	RNAseq (Illumina HiSeq 2000)	BCG 10 mg (2.10^7^ CFU); i.p.	7 days
Srinivasan et al. (2016)	Mouse	Cortices + Hippocampi	FCS	Cd11b^+^ staining	RNAseq (Illumina HiSeq 2500)	LPS 0111:B4 E.Coli (Sigma) 10 mg/kg; i.p.	24 h

Looking at the impact of BCG immune challenge at a more delayed time-point, Gonzalez-Pena et al. (2016) reported that, whereas recovery from the sickness behavior was already achieved 7 days after the inoculation, microglia/monocytes transcriptome dysregulation was still not resumed. Indeed, at this time-point, a large proportion of genes were still deregulated and functional analysis highlighted the enrichment of categories such as “immune response” and “chemotaxism”. Interestingly, their analysis also pointed to the deregulation of more specific pathways such as tryptophan or inositol metabolism that have been associated with depression-like behaviors (Dantzer et al., 2008).

### Gliomas

Gliomas include different types of glial tumors (astrocytoma, oligodendroglioma and glioblastoma). Depending on the cell type and aggressiveness, gliomas are graded from I (relatively benign) to IV (or glioblastoma); glioblastoma being the most common aggressive tumor of the CNS presenting a median survival of only 15 months (Thakkar et al., 2014). Microglia or macrophages constitute 30%–50% of the glioma cells, indeed, gliomas are composed of both neoplastic and non-neoplastic cells that form tumors and participate to cancer progression (Hambardzumyan et al., 2016). Tumor-associated macrophages (TAMs) either originate from CNS parenchyma (i.e., microglia) or from the periphery and both compose numerous non-neoplastic cells. TAMs not only release growth factors and cytokines but also facilitate neoplastic cell expansion and migration thus facilitating tumors proliferation and survival. Alike their malignant counterparts, most astrocytomas contain TAMs.

In a recent study, Szulzewsky et al. (2015) transplanted a glioma cell line of murine origin in the brain of CX3CR1^+/eGFP^ Ccr2^RFP/wt^ mice (to discriminate between microglia and peripheral monocytes) and carried out transcriptomic analyses. Twenty days post-injection, microglia/macrophages were obtained by magnetic-activated CD11b^+^-mediated flow cytometry and gene profile was obtained by microarray (for details see Table 1). Setting a threshold of at least 2-fold, 783 and 198 genes were identified as up and down-regulated, respectively in a heterogeneous population of glioma-associated microglia/macrophages (GAMs) as compared to naive microglial cells. GO enrichment analysis highlighted as most enriched pathways, “regulation of immune response/activation”, “programmed cell death”, and “response to other organism/to virus” (Supplementary Table S1). Comparison of GAMs de-regulated genes to those of polarized macrophages (M1 or M2a, b, and c phenotypes) showed only partial overlap. Indeed, 59.6% of the up-regulated genes in GAMs were not up-regulated in the four macrophage groups.

The authors also demonstrated that *Gpnmb* and *Spp1*, as in murine GAMs, were highly up-regulated in the human samples from both glioblastoma and lower grade brain tumors as compared to control (Szulzewsky et al., 2015). Moreover, high expressions of these genes were associated with poor survival outcomes.

Using RNA-Seq, the same group also compared expression profiles of CD11b^+^ GAMs from human and CD11b^+^ microglia from non-tumor human samples (Szulzewsky et al., 2016). Three-hundred and thirty-four genes presented a 2-fold or greater difference between conditions and GO analysis identified genes associated with “mitotic cell cycle”, “cell migration”, “cell adhesion” and “extracellular matrix organization” as the most enriched in the GAMs samples (Supplementary Table S1). Comparison of mouse and human GAMs highlighted that “mitotic cell cycle” and “extracellular matrix organization” de-regulation was shared by both species. However, surprisingly, and in contrast to murine GAMs, human GAMs did not display an up-regulation of pathways related to immune activation such as “immune system” and “cytokine signaling”.

## What Specific Microglia Transcriptomes Analyses Taught and did Not Teach us on Microglia Reactivity?

In the whole tissues, gene expression analysis represents each gene’s average expression among all cells and does not allow the identification of the cell type(s) responsible for a gene’s physiological expression or its altered expression in pathological condition. Additionally, if changes in gene expression are restricted to a specific cell type, modification may be too small to be identified using the whole tissues. Alternatively, increase and decrease in gene expression in the whole tissue may respectively results from cell proliferation and cell death, or changes in gene expression at the cell level. Information relative to changes in specific cell population is thus critical to understand their roles in the CNS pathologies and to design targeted therapeutic strategies. Cell-specific transcriptomic studies overcome this limitation and allow a better understanding of the mechanisms involved in the different CNS pathologies initiation and progression.

Studies discussed in the present review clearly highlight that microglia activation is a multiple and complex phenomenon and that each pathological microglial state depends on the type of stimulus, its duration and severity, as well as its local brain environment. Time after the initial stimulus (i.e., acute conditions) or stage of disease development (i.e., chronic conditions) is also obviously a key factor in the remodeling of the microglial transcriptomic profile. All the above-mentioned parameters as well as environmental factors (including a local breeding conditions) have significant impacts on the microglia reactivity and may explain the variability observed. In addition to the biological conditions, multiple aspects can also influence microglia transcriptome determination including: (1) the experimental design (especially the number of replicates) which has direct influence on the number of deregulated genes as well as the technical approaches used to; (2) isolate microglia (from flow cytometry vs. microdissection, to the markers used for purification in FACS-based studies); (3) obtain transcriptomic data (microarrays vs. RNA-Seq); and (4) bio-informatically analyze the data. Methodologies may indeed also have significant impact on the final results. As a result of this diversity and of the specificity of the technical approaches used (especially for the less standardized RNA-Seq approaches), it is difficult to directly compare data from different studies.

Nevertheless, *in silico* comparisons of differentially expressed genes in comparable datasets (i.e., in the same animal model) lead to the identification of common genes and pathways among the different datasets, thus highlighting the power of cell-specific analysis to unbiasedly uncover relevant molecular pathways in the development of a given pathology (Figure 1).

Comparing such lists of differentially expressed genes (DEGs) is now possible through the Glia Open Access Database (GOAD^1^), which contains a growing collection of transcriptomic datasets of glial cells (including microglial cells) both in homeostatic and pathological conditions (Holtman et al., 2015a). Such tool may be useful to determine whether a core of microglial genes deregulated in many if not all pathological conditions could be identified. Such set of genes would represent useful biomarkers to study the involvement of microglia in any CNS pathological conditions and could be used to monitor the effects of therapeutic strategies. Consistent with this hypothesis Keren-Shaul et al. (2017) identified a similar microglia subpopulation between mouse models of ALS and AD. This suggests that DAMs are not specific to a particular disease etiology or stage but rather are associated with the general program involved in the clearance of misfolded and/or aggregated proteins, a common feature to many different neurodegenerative conditions.

Differences and similarities in microglia reactivity in several pathological conditions can also be studied at the level of the deregulated biological processes. To meet this goal, we performed a GO-based analysis of available DEGs lists using the freely available Panther software (Mi_2017^2^; GO-slim biological process) and reanalyzed 14 different datasets arising from eight different studies (Chiu et al., 2013; Orre et al., 2014a; Holtman et al., 2015b; Noristani et al., 2015, 2017; Szulzewsky et al., 2015, 2016; Bennett et al., 2016). Using the same analysis tool allows a more accurate comparison of the deregulated biological processes and highlights commonly deregulated processes in both neurodegenerative and non-neurodegenerative pathological conditions (Supplementary Tables S1, S2). Drawing conclusions from such comparisons is difficult since not all pathological conditions were studied longitudinally. Nevertheless, one common feature emerging from this analysis is that cell proliferation seems to occur in the early phases of microglial reactivity whereas inflammatory processes develop later.

Although useful, GO-based categorization may provide an over-simplistic view of the actual de-regulated pathways and of the associated functions. Indeed, genes, and this is particularly true for immune-related transcripts, may play different roles depending on the context. As an example, TNFα, which is primarily categorized as a pro-inflammatory molecule is also known to play an important role in “synaptic scaling”, a form of synaptic plasticity (Stellwagen and Malenka, 2006). These findings led to the recent challenging of the concept of microglia as a component of innate immunity and propose that dysfunction heterogeneity of microglia in diseases may be better reflected by the contribution of multiple pathways called “system biology” rather than conventional proinflammatory and/or anti-inflammatory models (Masgrau et al., 2017). It is also important to keep in mind that the GO-based categorization present limitations including the fact that many genes are not annotated in ontological databases and may not reflect microglia functions in physiological and pathological conditions.

Although not always straight-forward to use for a typical biologist, other specific bioinformatics analyses, and in particular co-variation studies, certainly represent efficient tools to compare the transcriptomic alterations in various pathological conditions. In this respect, Holtman et al. (2015b) have recently used Weighted Gene co-expression Network Analysis to compare transcriptomes of microglia in aging, AD and ALS mouse models. In addition to disease-specific microglial signatures, their analyses identified common transcriptional profiles for up-regulated genes in the different neurodegenerative conditions. Key features of this profile were related to “phagosomes”, “lysozyme”, “antigen presentation” and “AD signaling”. Interestingly, the analyses also revealed that acute microglial activation induced by LPS led to a rather different transcriptomic signature in which NK-κB signaling played a central role. The development of such analyses might help drawing the commonalities and singularities of microglial activation in the different pathological conditions, and may thus help designing better targeted therapeutic strategies.

In regards to the published data, the remodeling of the microglia transcriptome in a given pathology has been studied in rather restricted numbers of animal models (Table 1). In the objective of translation to the clinics, it would be important to verify: (1) if deregulated genes in a given animal model is also deregulated in another animal model of the same disease from the same species; and (2) ultimately to investigate the involvement of identified genes in human. Importantly, microglia/macrophages-specific gene profiling has shown that despite differences between animal models and human diseases, candidate genes identified in rodent models of a given pathology were also de-regulated in human pathological samples. It thus attests that cell-specific transcriptomic analysis is not only a powerful approach to decipher molecular mechanisms but also to identify precise therapeutic strategies.

## Futures Directions to Study Microglia Transcriptomic Changes Under Pathological Conditions

So far, under each specific experimental condition (i.e., in a given tissue and at a given stage of the disease progression), pathological microglia have only been studied as a global cell population. However recent transcriptomic studies have clearly enlightened that homeostatic microglia are indeed diverse. First, Hickman et al. (2013) and Orre et al. (2014a), using RNA-Seq and microarray approaches respectively, have identified specific microglial changes in gene expression during normal aging. More recently, isolating microglia from four different brain regions (cortex, striatum, hippocampus and cerebellum) at different age (4, 12 and 22 months old mice) Grabert et al. (2016) have shown that adult microglia display transcriptional identities with different function depending on the brain region and age. In a recently study, microglia were isolated from different human brain regions via surgical resection to identify a microglial gene signature including 881 transcripts. It also highlighted that many genes enriched in microglia displayed diverse modifications in expression upon neurodegenerative contexts and upon transfer to an *in vitro* environment. Thus, emphasizing other limitations of studying microglia (Gosselin et al., 2017). These results are in agreement with the current view that microglia are immune competent cells that are tightly adapted to their local environment (Hanisch, 2013; Wolf et al., 2017).

Interestingly, using a global and single cell RNA-Seq approach (Matcovitch-Natan et al., 2016) revealed that during brain development diverse microglial cell populations can co-exist in a specific brain environment. Such diversity of the microglial cells under pathological conditions has already been demonstrated. For example, it has been shown that in AD brains microglia associated with amyloid plaques display histological signs of activation, including larger cell body and thicker and shorter ramifications. Similar findings apply to microglia recruited to CNS lesion sites. However, the specific transcriptomes of microglia associated to plaques or lesions vs. those located further away but within close proximity to the pathological zone have, to our knowledge, never been studied. The recent development of single cell RNA-Seq approaches now enable to unbiasedly tackle those questions. Indeed, combining this powerful approach with adequate bio statistical analyses will allow to unbiasedly classify cells located in a specific pathological CNS environment in various sub-populations and to determine their potential role in disease progression. The presence of distinct microglia subpopulations in a given pathological states may well explain why microglial cells have been shown to play indistinctly neutral, beneficial and deleterious effects in multiple pathologies. Such characterization might also help developing better targeted therapeutic strategies aimed at preventing the deleterious effects of microglial activation while boosting their beneficial ones. A remarkable example of such study has very recently been published by the groups of I. Amit and M. Schwartz (Keren-Shaul et al., 2017). As highlighted above, it led to the identification of a specific microglia sub-type that may be involved in restricting the disease progression in AD. Even more ambitious, is the concomitant transcriptomic profiling of several cell populations including neuronal and glial cells within the same pathological condition. This would certainly help to better understand the cellular cross talks between the different cell types under both physiological and pathological conditions.

## Author Contributions

HEH, HNN and FEP contributed to the writing of the manuscript. HEH and FEP gave the final approval.

## Conflict of Interest Statement

The authors declare that the research was conducted in the absence of any commercial or financial relationships that could be construed as a potential conflict of interest.

## Footnotes

^1^www.goad.education

^2^http://pantherdb.org

## Abbreviations

AD: Alzheimer’s disease
ALS: amyotrophic lateral sclerosis
BCG: bacille Calmette Guérin
CNS: central nervous system
EAE: experimental autoimmune encephalomyelitis
eGFP: enhanced green fluorescent protein
FACS: fluorescence-activated cell sorting
GAM: glioma-associated microglia/macrophages
GO: gene ontology
NGS: NextGen sequencing
LCM: laser capture microdissection
LPS: lipopolysaccharide
MS: multiple sclerosis
RNA-Seq: RNA sequencing
SCI: spinal cord injury
TAM: Tumor-associated macrophages
TBI: traumatic brain injury
TMEM119: Transmembrane Protein 119
WT: wild type.
